# Treatment Failure of Cerebral Venous Thrombosis With Rivaroxaban: A Case Report and Narrative Review

**DOI:** 10.7759/cureus.23778

**Published:** 2022-04-03

**Authors:** Mustafa Mohamed, Muzamil Musa, Abdalla Fadul, Ibtihal Abdallah, Mustafa Najim, Abuzar Saeed

**Affiliations:** 1 Internal Medicine, Hamad Medical Corporation, Doha, QAT; 2 Clinical Pharmacy, Hamad Medical Corporation, Doha, QAT

**Keywords:** rivaroxaban, dabigatran, cerebral vein thrombosis (cvt), venous thromboembolism (vte), : direct oral anticoagulants (doac)

## Abstract

Direct oral anticoagulants (DOACs) are approved for the treatment and prevention of venous thromboembolism (VTE), with multiple advantages they offer over the older available anticoagulant drugs. Cerebral vein thrombosis (CVT) is an uncommon type of stroke that is usually associated with intracerebral hemorrhage (ICH), for which early anticoagulation is still recommended to prevent thrombus expansion, to facilitate recanalization, and to prevent the development of deep vein thrombosis (DVT) and pulmonary embolism (PE). DOACs’ use for the treatment of CVT is an area of clinical equipoise, and hence it is still not recommended especially in the acute phase. This case report presents a patient who was on a therapeutic dose of rivaroxaban after an episode of CVT. He developed another CVT that was evident on an intracranial CT (CT) venogram. This was considered a therapeutic failure of DOACs, specifically rivaroxaban, in preventing the recurrence of CVT. Such observation might add to the existing yet scarce body of evidence regarding the use of DOACs in the anticoagulation management of CVT.

## Introduction

Direct oral anticoagulants (DOACs) are a class of drugs that work via the inhibition of factor Xa (rivaroxaban, apixaban, edoxaban, and betrixaban) or thrombin (dabigatran). These agents have been approved for the treatment and prevention of venous thromboembolism (VTE) in relatively recent years, starting with dabigatran which was the first DOAC to be approved in 2010. They offer a few advantages over the existing anticoagulants [e.g. the vitamin K antagonist warfarin, unfractionated heparin (UFH), and low molecular weight heparin (LMWH)]. These advantages include their oral route of administration, immediate onset of action, predictable pharmacokinetics and hence fixed dosing schedules and no need for monitoring, and less interactions with drugs and food items [1]. DOACs have become the mainstay of treating and preventing VTE when no contraindication for their use is present (e.g. coadministration with strong inhibitors or inducers of P-glycoprotein or cytochrome P450 enzymes) [2-3].

Cerebral vein thrombosis (CVT) is an uncommon type of stroke that affects about 0.5%-1% of all stroke patients [4]. It is more frequently encountered among younger patients less than 50 years of age, and its risk factors are diverse. Risk factors include infections, pregnancy, dehydration, use of oral contraceptive drugs, head trauma, or substance abuse [5]. Other medical conditions such as inflammatory bowel disease might predispose a patient to CVT as well [6].

CVT is commonly associated with intracerebral hemorrhage (ICH). Despite that, early anticoagulation is recommended to prevent thrombus expansion, to facilitate recanalization, and to prevent the development of deep vein thrombosis (DVT) and pulmonary embolism (PE) ([5-7].

The use of DOACs for the treatment of CVT is an area of clinical equipoise, and the available evidence is deemed weak and insufficient by international societies due to its observational nature and the high risk of associated bias. Due to such a lack of data, DOACs are still not recommended for treating CVT, especially in the acute phase [7]. Despite that, successful treatment of CVT with rivaroxaban has been reported in observational studies, but no robust evidence currently exists to confirm its efficacy or safety [8-9].

Here we report our experience of treatment failure and CVT recurrence with rivaroxaban. Up to our knowledge, this is the first case report of treatment failure with rivaroxaban in the setting of CVT.

## Case presentation

This is a case of a 47-year-old, 79-kg weighing man from an East Asian race. He has a past medical history of poorly controlled type 2 diabetes mellitus, with glycated hemoglobin (Hb A1C) of 10.5%. Additionally, our patient reported a brain surgery 12 years prior to his presentation to our hospital; however, he had no records of its details.

Upon his presentation to Hamad General Hospital in February 2020, he complained of dizziness and unsteady gait. Furthermore, he had two episodes of generalized tonic-clonic seizures in the Emergency Department, which were aborted with IV lorazepam. The patient had an urgent CT scan that showed left temporal venous hemorrhagic infarct secondary to thrombosis of the left sigmoid and transverse sinuses and vein of Labbe. It also showed surrounding perilesional edema with mass effect in the form of effacement of the overlying cortical sulci, compression of ipsilateral lateral ventricle, and partial effacement of the left ambient cistern. An initial thrombophilia screen was done and showed normal antithrombin III, factor V Leiden, and protein S. However, protein C activity was mildly reduced with a value of 60%. Additionally, a reduced activated protein C (APC) ratio was noted, which is suggestive of APC resistance. Screening for antiphospholipid antibody syndrome (APLS) was done and it showed negative anticardiolipin antibodies [immunoglobulin (Ig)G and IgM], negative anti-beta2-glycoprotein antibody (IgG and IgM), and negative lupus anticoagulant.

Left-sided decompressive craniectomy was done and the patient was started on heparin infusion, which was subsequently changed to enoxaparin at a therapeutic dose. On the 13th of March 2020, the anticoagulation was changed from enoxaparin to dabigatran at a dose of 150 mg orally twice daily, in line with the findings of the RE-SPECT CVT trial [9]. The plan by the attending medical team was to anticoagulate for six months with dabigatran and to assess the patient’s clinical condition by the end of this period. If clinically stable, protein C activity was to be repeated two weeks after the cessation of anticoagulation as the initially detected low protein C activity could be attributed to the acute condition.

**Figure 1:**
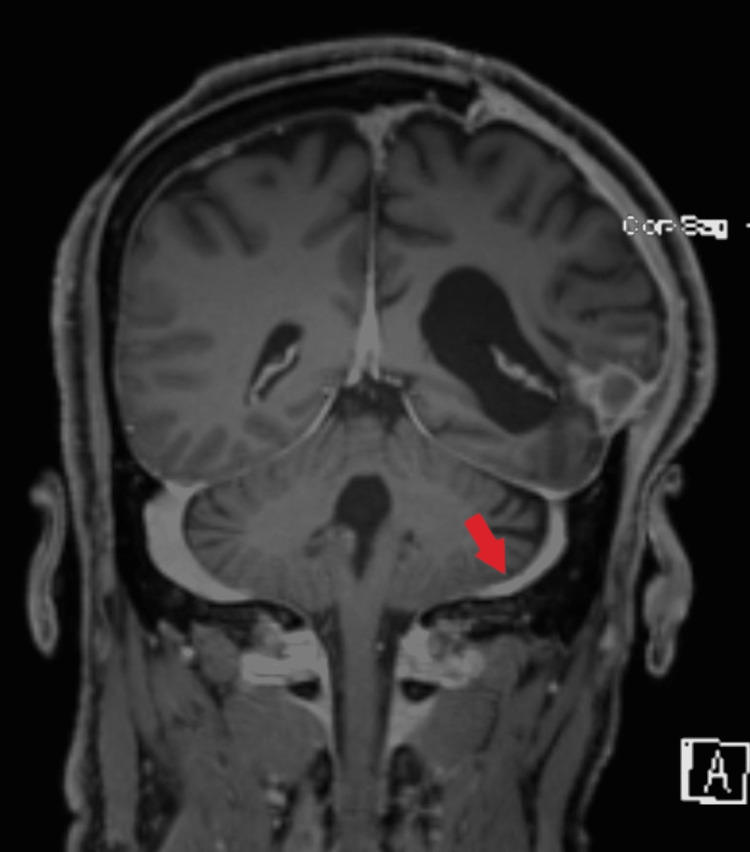
An MRI head: arrow showing re-canalization of the left sigmoid sinus.

An MRI head was done before transferring the patient to a long-term facility and showed evidence of re-canalization of cerebral venous sinuses (Figure 1). After transferring him to a long-term nasogastric tube (NGT) it was inserted to facilitate the administration of medications as the patient became agitated. Dabigatran was subsequently stopped as the capsules shall not be opened, and hence dabigatran cannot be administered via NGT. Rivaroxaban, which is more feasible to be crushed and administered through NGT, was started at a dose of 20 mg orally daily on the 15th of March 2020. 

**Figure 2:**
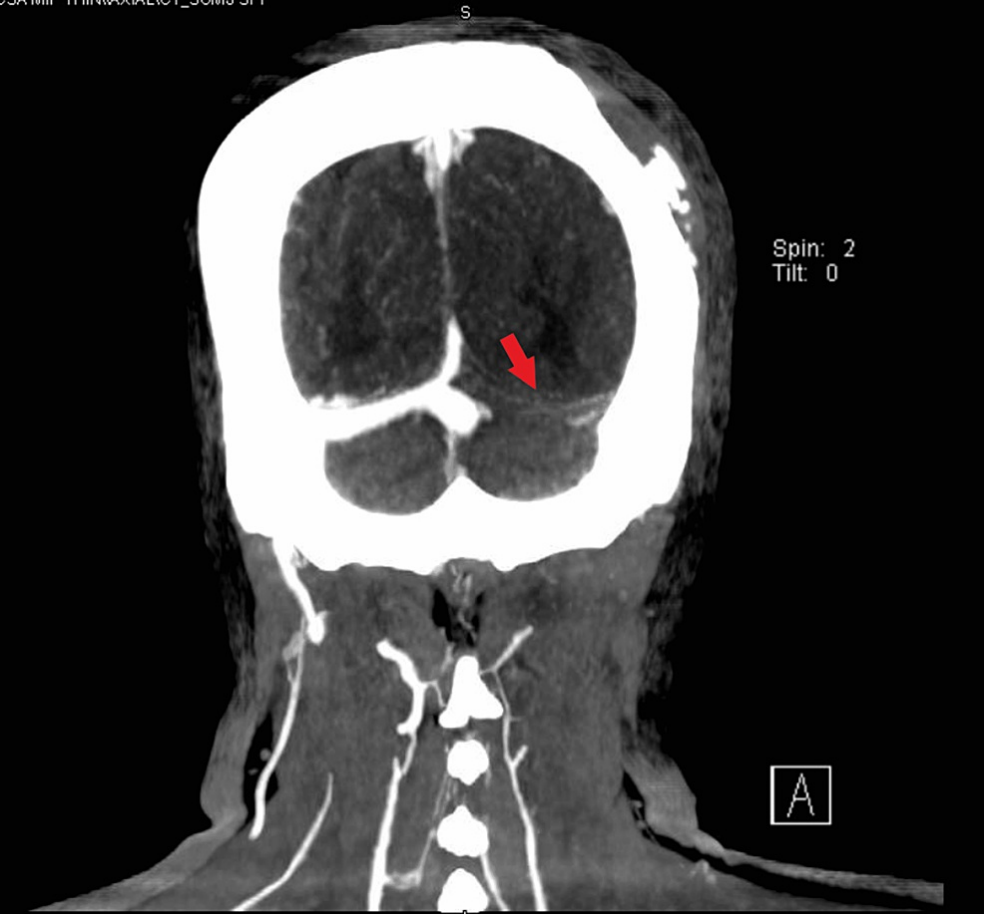
CT head venogram: arrow showing new filling defect in the left sigmoid sinus.

Our patient was symptom-free until June 29, when he started to be more agitated again yet he had no seizure or any other new focal neurological findings. An intracranial CT venogram was done and showed a newly seen suspicious filling defect in the left sigmoid sinus indicating a new CVT (Figure 2). Additionally, the incidental left temporal abscess was seen on MRI. Because this abscess was small in size and the patient was afebrile with normal inflammatory markers (white blood cells, C-reactive protein, lactic acid, and procalcitonin were all within normal ranges) and he had no new neurological deficit, our infectious diseases consultant as well as our neurosurgery consultant suggested IV antibiotics as treatment for it instead of surgical intervention was proven to be effective in his case later on. The occurrence of new CVT despite full anticoagulation with rivaroxaban was considered a treatment failure, and dabigatran was restarted on the 3rd of July 2020 through the oral route.

## Discussion

Cerebral venous thrombosis is a rare condition. Given its low prevalence, the evidence regarding the best therapeutic options is scarce and slowly evolving over years. The recommendations from international guidelines to use LMWH, UFH, or warfarin for CVT treatment have been unchanged for many years. Moreover, most of the available evidence comes from observational studies. The potential and inherited biases from the observational design limit the ability to make strong recommendations based on these studies.

The only randomized clinical trial that examined the use of DOACs in the treatment of CVT was the RE-SPECT CVT trial. It compared dabigatran at a dose of 150 mg twice daily to the international normalized ratio (INR)-adjusted warfarin for the treatment of CVT, at a follow-up period of 25 weeks. Among the 120 included patients, there were no recurrent VTEs observed in either of the two treatment groups. However, one case of major gastrointestinal bleeding occurred in the dabigatran group, while two cases of intracranial bleeding were reported in the warfarin group. The rate of recanalization was similar across the groups as well: 33 patients in the dabigatran group (60.0%; 95% CI, 45.9-73.0) and 35 patients in the warfarin group (67.3%; 95% CI, 52.9-79.7). RE-SPECT CVT suggests that dabigatran might be a safe and effective alternative for warfarin in the management of CVT. As discussed earlier, these findings have not yet been incorporated into clinical practices guidelines [9].

Rivaroxaban, like other DOACs, has not yet been endorsed by the international societies for the treatment of CVT. Nonetheless, Rivaroxaban has been utilized on an individual basis to treat CVT in clinical practice, and some case reports have reported successful outcomes [8]. Up to our knowledge, there is no reported treatment failure of CVT with rivaroxaban in the literature yet.

In the case of our patient, we hypothesize that rivaroxaban has failed in preventing the recurrence of CVT. The brain abscess that was observed concurrent with the second CVT diagnosis could be a rare complication of venous infarct, and it could possibly be the provoking event for the thrombosis [10]. However, since rivaroxaban was used in its full therapeutic dose, we believe that this should serve as a sufficient preventative measure for any VTE.

## Conclusions

Our case report adds to the uncertainty regarding the use of DOACs in the treatment of CVT. In the absence of well-designed multi-center randomized controlled trials, no strong recommendation can be made in favor of or against the use of DOACs in this patient population.
